# Kinetics and Mechanism of the Reaction between Chromium(III) and 3,4-Dihydroxy-Phenyl-Propenoic Acid (Caffeic Acid) in Weak Acidic Aqueous Solutions

**DOI:** 10.1155/2008/624583

**Published:** 2008-07-28

**Authors:** Vladimiros Thoma, Konstantinos Tampouris, Athinoula L. Petrou

**Affiliations:** Laboratory of Inorganic Chemistry, University of Athens, Panepistimioupolis, Athens 15771, Greece

## Abstract

Our study of the complexation of 3,4-dihydroxy-phenyl-propenoic acid by chromium(III) could give information on the way that this metal ion is available to plants. The reaction between chromium(III) and 3,4-dihydroxy-phenyl-propenoic acid in weak acidic aqueous solutions has been shown to take place by at least three stages. The first stage corresponds to substitution (*I*_d_ mechanism) of water molecule from the Cr(H_2_O)_5_OH^2+^ coordination sphere by a ligand molecule. A very rapid protonation equilibrium, which follows, favors the aqua species. The second and the third stages are chromium(III) and ligand concentration independent and are attributed to isomerisation and chelation processes. The corresponding activation parameters are Δ*H*_2(obs)_^≠^ = 28.6 ± 2.9 kJ mol^−1^, Δ*S*_2(obs)_^≠^ = −220 ± 10 J K^−1^mol^−1^, Δ*H*_3(obs)_^≠^ = 62.9 ± 6.7 kJ mol^−1^ and Δ*S*_3(obs)_^≠^ = −121 ± 22 J K^−1^mol^−1^. The kinetic results suggest associative mechanisms for the two steps. The associatively activated substitution processes are accompanied by proton release causing pH decrease.

## 1. INTRODUCTION

**Figure 1:**
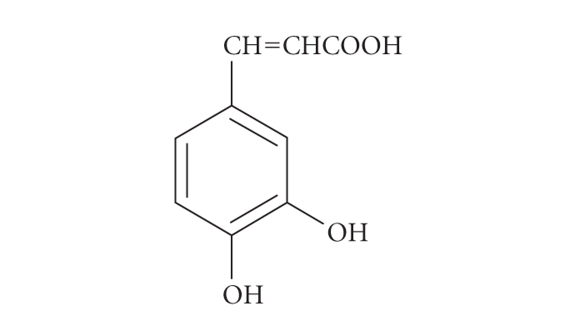
Caffeic acid.

Polyphenols are widely found in plants and are present in all plant-derived systems [1–3]. Hydroxy-cinnamates, especially caffeic acid (see Figure 1) and its derivatives, are widely distributed and their presence in fruit juices is due to their easy extractability. Foods containing polyphenols undergo enzymatic and nonenzymatic browning due to autoxidation reactions [4, 5].

Initial oxidation of compounds like caffeic acid results in the formation of their quinone form [6–8], which, being strongly electrophilic, undergoes nucleophilic attack [9–11]. The rest of the reaction in which the quinone participates is the same, no matter if the quinone has been produced enzymatically or chemically.

The above reaction takes place under acidic conditions and is catalysed in the presence of metals [12–14]. Food processing that includes treatment in alkaline conditions results in decreased nutritional value due to phenol oxidation reactions with amino acids and proteins through their nitrogen [15].

Under alkaline conditions, caffeic acid reacts rapidly with oxygen [16]. Under acidic conditions, the nonenzymatic reaction is slow [17].

Researchers [18] have found that the oxidation of caffeic acid is extremely rapid at pH ≥ 8 and the pH dependency along with the relatively low and constant Arrhenius activation energies at all pH's indicate that the phenolate ions are also involved in the reaction.

The rate of the reaction is increased by increasing pH (4.56 × 10^−7^ s^−1^ at pH 4.0 and 1.67 × 10^−5^ s^−1^ at pH 8.0, both at 35°C) and temperature (2.12 × 10^−6^ s^−1^ at 5°C and 1.67 × 10^−5^ s^−1^ at 35°C, both at pH 8.0; 1.39 × 10^−7^ s^−1^ at 5°C and 2.29 × 10^−6^ s^−1^ at 35°C, both at pH 5.0) [18]. The Arrhenius activation energy was found to be 49.0 ± 6.4 kJ mol^−1^ at pH 8.0 and the phenolate anion concentration was proposed to be the controlling factor of the rate of autoxidation [18].

Our goal was to study the kinetics and mechanism of the complexation of caffeic acid with chromium(III) under weak acidic aqueous conditions that exclude the autoxidation of the ligand.

Caffeic acid, being a degradation product of humic acids, can be found in soils and nutrient solutions where plants grow. The study of the complexation of caffeic acid by metal ions, in our case by chromium(III), could give information on the way that metal ions are available to plants.

## 2. EXPERIMENTAL RESULTS

### 2.1. Reagents and materials

The reagents used were all of analytical grade. Caffeic acid (Alfa Aesar), used as received, was dissolved in dilute (0.1 M) solution of KOH in concentrations ranging from 0.0025 to 0.01 M. Stock solutions (0.2 M) of Cr(III) were prepared from Cr(NO_3_)_3_·9H_2_O. The ionic strength was adjusted using KNO_3_. The solutions were used immediately after their preparation to avoid transformation and decomposition reactions. The addition of the chromium(III) solution kept the pH below 4 due to its acidic hydrolysis. All kinetic experiments were performed at pH values below 4 in the presence of air. At 21°C, the starting pH of the solutions was 2.3.

### 2.2. Kinetic experiments

The kinetics of the substitution of water molecules in the coordination sphere of Cr(H_2_O)_6_^3+^ by caffeic acid were followed at 537 nm, where the biggest absorbance difference between the final product and the initial (*t* = 0) mixture of Cr(H_2_O)_6_^3+^/caffeic acid exists. The absorbances were recorded on a Hitachi Model 100-60 spectrophotometer and the electronic spectra were recorded on a Varian Cary 3E spectrophotometer.

First-order rate constants were estimated with a nonlinear least-squares fit.

Pseudofirst-order conditions (excess of Cr(III), i.e., acidic solution) were employed for all of the kinetic experiments, whereas experiments in excess of ligand (caffeic acid) were not possible to be performed due to its oxidation in the alkaline solution of the reaction mixture. Experiments at temperatures higher than 45°C were also avoided due to the acceleration of the ligand's decomposition/autoxidation.

**Figure 2:**
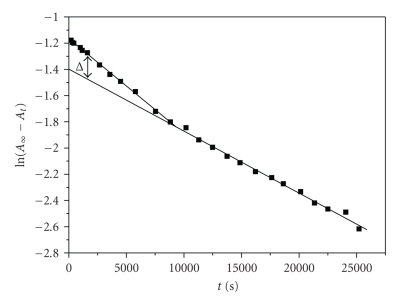
A typical nonlinear plot of ln(*A*_∞_ − *A*_*t*_) versus time. Conditions: [caffeic acid]_0_ = 0.01 M, [Cr(III)]_0_ = 0.08 M, 1 cm cell, *T* = 303 K, *I* = 0.5 M.

The plots of ln(*A*_∞_ − *A*_*t*_), where *A*_∞_ and *A*_*t*_ are absorbances after the completion of the reaction and at time *t*, against time were found to be nonlinear (see Figure 2); they are curved at short reaction times and have constant slope at large reaction times.

The rate constants were calculated assuming two consecutive first-order steps according to methods found in the literature [19, 20].

A reaction sequence consisting of two first-order (or pseudofirst-order) steps

(1) $$\text{A}\overset{k_{2}}{⟶}\text{B}\overset{k_{3}}{⟶}\text{C}$$

admits of two mathematical solutions; the sets of rate constants are such that the fast and slow kinetic steps are interchanged [19, 21, 22].

**Figure 3:**
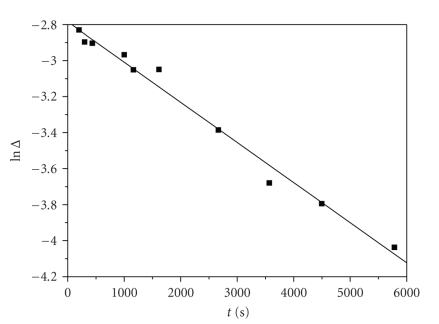
A typical plot of lnΔ versus time. Conditions: [caffeic acid]_0_ = 0.01 M, [Cr(III)]_0_ = 0.08 M, 1 cm cell, *T* = 303 K, *I* = 0.5 M.

The linear second part (long-time) gives a slope of −*k*_3_ and thus the *k*_3(obs)_ values were obtained. The rate constants *k*_2(obs)_ for A → B step were evaluated by the method of Weyh and Hamm [20] using the consecutive rate equation

(2) $$A_{\infty }\;-\;A_{t}\;=\;a_{2}e^{-k_{2\left(\text{obs}\right)}t}\;+\;a_{3}{\text{e}}^{-k_{3\left(\text{obs}\right)}t},$$

where *a*_2_ and *a*_3_ are dependent upon the rate constants and the extinction coefficients. The values of *a*_2_ *e* ^−*k*_2(obs)_*t*^ were obtained from Δ = *A*_∞_ − *A*_*t*_ − *a*_3_ *e* ^−*k*_3(obs)_*t*^ at various times *t* (see Figure 2). So, lnΔ = constant − *k*_2(obs)_ *t* and the *k*_2(obs)_ values were derived from the slope of the plots of lnΔ versus *t* for small values of *t*. A typical plot is shown in Figure 3.

The experimental data show a curvature of the ln(*A*_∞_ − *A*_*t*_) versus *t* plot at all temperatures for various (excess) chromium(III) concentrations. The assumption of the existence of two consecutive steps for the under-study reaction and the computation of *k*_2(obs)_ and *k*_3(obs)_ values fits well with the experimental data. The analysis of the data gives a small value for Δ*H*_2(obs)_^≠^ and a very big absolute value for Δ*S*_2(obs)_^≠^ suggesting that a composite reaction takes place; this could be a fast equilibrium followed by the *k*_2_ slow step [19]. Contribution of uncomplexed Cr(III) species in the absorbance values, mainly due to the excess of Cr(III), does not cause problems in the graphs (see Figure s 2 and 3) because it is included in both *A*_∞_ and *A*_*t*_ and is eliminated due to the subtraction of *A*_*t*_ from *A*_∞_.

Table 1 gives the *k*_2(obs)_ and *k*_3(obs)_ values for the reaction at various temperatures (298 K, 303 K, 308 K, and 310 K).

**Figure 4:**
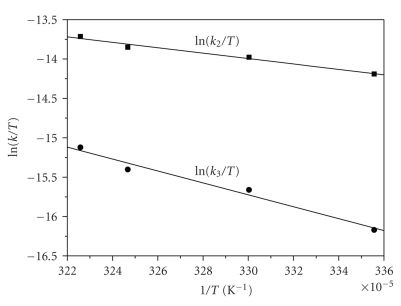
The Eyring plots for *k*_2(obs)_ and *k*_3(obs)_ at the temperatures studied.

The activation parameters Δ*H*_2(obs)_^≠^, Δ*S*_2(obs)_^≠^ and Δ*H*_3(obs)_^≠^, Δ*S*_3(obs)_^≠^, corresponding to *k*_2(obs)_ and *k*_3(obs)_, respectively, are calculated from the linear Eyring plots (see Figure 4) and are given in Table 2.

At times longer than four to five half-lives of the B → C step, a reaction involving oxidation of the ligand sets in, causing anomalous further absorbance changes. The recorded values of *A*_∞_ used for Figures 2, 3, and all other graphs were very close to the true values since they were also obtained by plotting *A* = *f* (*t*); it was thus possible to confirm the completion of the reaction.

## 3. DISCUSSION

The value of pKa for Cr^3+^/Cr(OH)^2+^ is about 4 [23, 24]. At pH < 4, chromium(III) complex exists mainly in its hexa-aqua monomeric form with absorbance maxima at 575 and 410 nm. However, over the pH range 3-4 there is some Cr(H_2_O)_5_OH^2+^ and the reaction with Cr(H_2_O)_5_OH^2+^ rather than with Cr(H_2_O)_6_^3+^ should be considered as taking place because of its largely higher reactivity. All the experiments in this work were conducted at pH values lower than 4. Under these conditions the ligand exists mainly as a neutral molecule and a monoanion [25, 26].

**Scheme 1:**
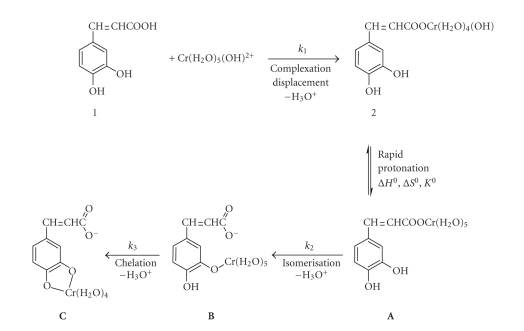


Upon mixing of the reactants, an initially violet (Cr_aq_^3+^) and a brown (caffeic acid) solution, a brown-green complex, is formed. The brown-green complex was formulated as an oxygen-bound chromium(III) compound **A** (Scheme 1) based on the UV/Vis spectra and the formation and subsequent transformation (substitution) kinetics. In all the kinetic experiments, we assume to begin with the already associated brown-green first complex **A**. This suggests that the following two slow steps (*k*_2_, *k*_3_) did not have a contribution on the first step at the temperatures studied.

The ln(*A*_∞_ − *A*_*t*_) versus time plots, being nonlinear, are indicative of a complex reaction, not a single-step process. The two first-order consecutive steps (analysis described above) were found to be independent on chromium(III) concentration (see Table 3), suggesting that a (first) step resulting in the formation of a complex upon reaction between the reactive forms of the reactants (Cr(H_2_O)_5_OH^2+^, neutral form of caffeic acid) took place. Thus three steps at least (*k*_1_, *k*_2_, *k*_3_) take place (Scheme 1): the first, a fast one, which was not studied, and the last two, which result from the analysis of our data.

The fact that a first fast step is taking place with a Cr(III) reacting species is not consistent with substitution rate constants of the hexa-aqua form of chromium(III); thus the mechanistic pathway (Scheme 1) is suggested to most probably be through the hydroxy species.

**Figure 5:**
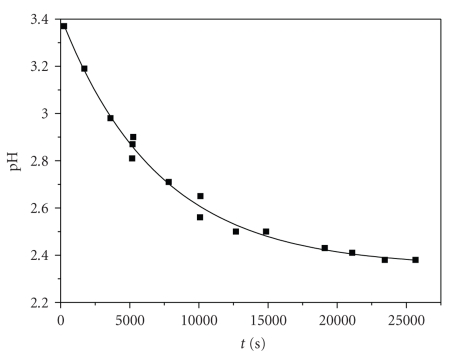
The pH versus time plots of a typical mixture of caffeic acid/Cr(III). Conditions: [caffeic acid]_0_ = 0.01 M, [Cr(III)]_0_ = 0.09 M, *T* = 308 K.

Because of the protonation of the ligand, the hydroxyl and the carboxylic groups are blocked, and the attacks by chromium(III) can take place on them by releasing protons, effect that is measured as pH decrease of the solution (see Figure 5). Experiments in excess ligand were not performed as explained above (Experimental Section 2) due to solubility problems and oxidation of the ligand in alkaline solutions.

The proposed mechanism for the formation of the various species is shown in Scheme 1. The attack of Cr(H_2_O)_5_OH^2+^ at the carboxylic group of caffeic acid, **1**, leads to complex **2**; this results in shifting equilibrium

(3) $${\text{Cr}({\text{H}}_{2}\text{O}{)}_{6}}^{3+}\overset{K_{a}}{⇌}\text{Cr}({\text{H}}_{2}\text{O}{)}_{5}{\text{OH}}^{2+}\;+\;{\text{H}}^{+}\;\text{pH}\;<\;4$$

to the right.

A very rapid protonation equilibrium which follows step 1 favors the aqua species (complex **A**). In conjugate-base mechanisms the conjugate base, though present as only a small fraction of the total, reacts and then reprotonates as it naturally would. The reactive species must be Cr(H_2_O)_5_OH^2+^ and not Cr_aq_^3+^.

Complex **A** reacts in two consecutive steps (*k*_2_, *k*_3_) to give **B** and **C**.

**Figure 6:**
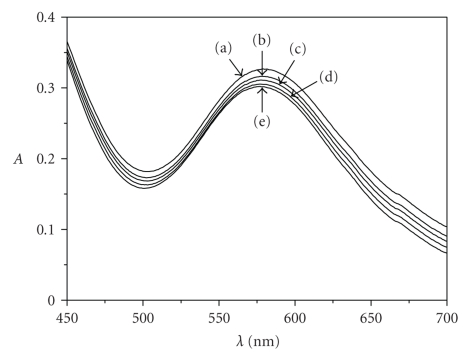
Spectra of a typical caffeic acid/Cr(III) mixture at various times after mixing; [caffeic acid]_0_ = 0.015 M, [Cr(III)]_0_ = 0.025 M, *T* = 298 K, 1 cm cell. (a) 0 seconds, (b) 1800 seconds, (c) 3600 seconds, (d) 7200 seconds, (e) 14400 seconds.

In Figure 6, spectra of the reaction mixture at 298 K are recorded at various times after mixing starting from the brown-green complex **A**. The spectra correspond to mixtures of **A**, **B,** and **C**.

The biggest absorbance difference between the spectra of **A** and **C** was found to be at 537 nm (Experimental Section 2). As a result of the complexation, the absorbance maximum was displaced to lower wavelengths (stronger ligand field, higher D_*q*_).

The *k*_2(obs)_ and *k*_3(obs)_ dependence on chromium(III) concentration (see Table 3) was studied at various temperatures in order (a) to find if a second or third chromium(III) ion is reacting with the already formed complex **A** and (b) to be able to calculate the activation parameters (Δ*H* ^≠^, Δ*S* ^≠^). By isolating the Cr^3+^/caffeic acid final complex (complex **C**) in solid form from Cr(III)/caffeic acid mixtures of various stoichiometries, we found that only one ligand molecule enters the coordination sphere of the metal. A stoichiometry of 1:1 for the reaction of Cr(III) with caffeic acid is proposed in accord with the elemental analyses and the consecutive first-order isomerisation and chelation reactions in the Cr(III) center. The elemental analyses: 28.11% C and 5.05% H correspond to the formula [Cr(H_2_O)_4_(caffeic acid_−3H_) K^+^OH^−^·2H_2_O] for which the calculated percentages for C and H are 27.49 % and 4.62%, respectively.

The assumption of two consecutive steps suggesting transformation of **A** to **B** and of **B** to **C** could be checked by the above studies. The independence of *k*_2(obs)_ and *k*_3(obs)_ on chromium(III) concentration confirms the above assumption.

The calculated activation parameters (Δ*H* ^≠^, Δ*S* ^≠^) obtained from the Eyring plots (temperature dependence experiments, Figure 4) suggest structures of the activated complexes formed in the corresponding processes (steps 2 and 3) as well as the mechanisms taking place.

It was not possible to obtain the activation parameters Δ*H*_1(obs)_^≠^ and Δ*S*_1(obs)_^≠^ since the wide range variation of temperature needed for the above purpose could not be achieved in our experiments. A dissociative mechanism *I*_*d*_ is, however, expected for step 1, since for the conjugate base Cr(H_2_O)_5_OH^2+^ dissociative mechanism *I*_*d*_ is supported [27] because the strong labilizing effect of the coordinated OH^−^ on the *trans* H_2_O leads to a 10^2^-10^3^ fold rate enhancement for the hydroxy-over the hexa-aqua ion.

The values of the activation parameters for step 2 (Δ*H*_2(obs)_^≠^, Δ*S*_2(obs)_^≠^) are implying that a composite reaction takes place. This consists of the fast equilibrium (*K*_0_) followed by the slow step 2 (Scheme 1). These composite activation parameters are Δ*H*_2(obs)_^≠^ = Δ*H* ^0^+Δ*H*_2_^≠^ and Δ*S*_2(obs)_^≠^ = Δ*S* ^0^+Δ*S*_2_^≠^, where Δ*H* ^0^ and Δ*S* ^0^ correspond to the equilibrium (*K* ^0^) and Δ*H*_2_^≠^ and Δ*S*_2_^≠^ to the second step (*k*_2_).

The values of Δ*H*_3(obs)_^≠^ and Δ*S*_3(obs)_^≠^ correspond to a single step *k*_3_.

The negative values of Δ*S* ^≠^ (Δ*S*_2(obs)_^≠^ and Δ*S*_3(obs)_^≠^), the independence of *k*_2(obs)_ and *k*_3(obs)_ on chromium(III) concentration, the displacement of *λ*_max_, and the pH decrease (see Figure 5) lead to the mechanism presented in Scheme 1, where substitution of water molecules in the coordination sphere of Cr(III) by caffeic acid molecule is associatively activated. The first step (*k*_1_) is an *I*_*d*_ replacement of water molecule by caffeic acid. The second step is an isomerisation and the third step is a chelation. All three steps take place with concomitant proton release. A stoichiometry of 1:1 for the reaction of Cr(III) with caffeic acid is proposed in accord with the elemental analyses and the consecutive first-order isomerisation and chelation reactions in the Cr(III) center.

The pH decrease (see Figure 5) is due to the release of protons upon the course of the reactions (Scheme 1).

**Scheme 2:**
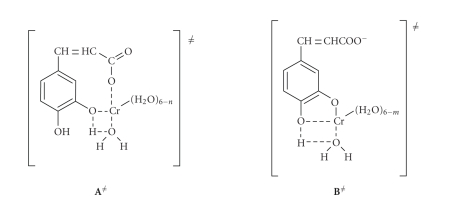
Activated complexes **A** ^≠^ and **B** ^≠^.

Structures of the activated complexes **A** ^≠^ and **B** ^≠^ are given in Scheme 2.

The suggested chelate formation in the transition states in Scheme 2 could occur preferably by an associative mechanism (Δ*S* ^≠^ < 0) which has been found to be operative in reactions of Cr(III) [28–30]. Alternatively, the original complex **A** could be a chelate itself, and this could explain the formation of products containing Cr–O bonds. The negative entropies of activation, Δ*S* ^≠^, however, suggest the formation of more organised transition states from the less well-organised reactants. Thus, complex **A** is not in chelated form and the mechanism shown in Scheme 1 is preferred. In this mechanism the phenolic groups act as internal attacking groups to the chromium-bound H_2_O molecules, which are thus supplied with a proton in the same complex (Scheme 2) and released as H_3_O^+^.

An alternative structure for the final product **C** is chelation at the carboxylic group through the oxygens. However, if chelation at the carboxylic group took place, a four-membered ring would result, which is less preferable. The five-membered ring which is supported to be formed according to the suggested mechanism (Scheme 1) is more probable. Thus we discard this alternative.

The two-step isomerisation—chelation of the first complex that is formed, **A**, produces PhO-bound chromium(III) species, characterised by UV/Vis spectra and kinetic behaviour which are typical of other known complexes containing Cr–O bonds [28, 29].

The above presented data suggest that binding is taking place through the two phenolic groups of the ligand molecule, caffeic acid, **1**. Such mode of binding (catecholic type) has been also suggested in the reaction of dihydrocaffeic acid with chromium(III) in weak acidic aqueous solutions [31]. The above reaction also takes place in at least three stages (complexation, isomerisation, chelation).

## Figures and Tables

**Table 1:** Values of *k*_2(obs)_ and *k*_3(obs)_ at various temperatures.

*k*_2(obs)_× 10^4^ (s^−1^)	*k*_3(obs)_× 10^5^ (s^−1^)	*T* (K)
2.0	2.8	298
2.6	4.8	303
3.0	6.3	308
3.4	8.4	310

**Table 2:** Activation parameters for steps $\text{A}\overset{k_{2}}{\to }\text{B}$ and $\text{B}\overset{k_{3}}{\to }\text{C}$.

ΔH_2(obs)_^≠^ (kJ mol^−1^)	ΔS_2(obs)_^≠^ (J K^−1^mol^−1^)	ΔH_3(obs)_^≠^ (kJ mol^−1^)	ΔS_3(obs)_^≠^ (J K^−1^mol^−1^)
28.6 ± 2.9	−220 ± 10	62.9 ± 6.7	−121 ± 22

**Table 3:** Dependence of *k*_2(obs)_ and *k*_3(obs)_ on Cr(III) concentrations at various temperatures for the reactions $\text{A}\overset{k_{2}}{\to }\text{B}\overset{k_{3}}{\to }\text{C}$. Conditions: [caffeic acid]_0_ = 0.005 M, *I* = 1.0 M.

*k*_2(obs)_× 10^4^ (s^−1^)	*k*_3(obs)_× 10^5^(s^−1^)	[Cr(III)]_0_(M)	*T* (K)
2.0	2.8	0.018	298
2.2	4.8	0.08	303
2.4	5.1	0.09	303
2.8	4.7	0.10	303
2.9	4.5	0.11	303
2.8	6.0	0.07	308
3.2	6.6	0.09	308
3.3	9.1	0.08	310
3.7	8.5	0.08	310
3.3	7.6	0.14	310
